# An Emerging Human Pathogen: Raoultella Planticola

**DOI:** 10.7759/cureus.92875

**Published:** 2025-09-21

**Authors:** Daniela Pi-Noa, Cinthia Reyes, Nikhil Sikha, Anita Sikha

**Affiliations:** 1 Internal Medicine, University of Miami at Holy Cross, Fort Lauderdale, USA; 2 Medicine, Ross University School of Medicine, Miramar, USA; 3 Internal Medicine, University of Miami Miller School of Medicine, Fort Lauderdale, USA

**Keywords:** immunocompromised, klebsiella spp, raoultella planticola, recurrent urinary tract infection, urinary tract infection

## Abstract

*Raoultella planticola (R. planticola)* is a gram-negative environmental bacterium rarely implicated in human infections. Urinary tract infections (UTIs) caused by *R. planticola* remain rare, particularly in outpatient settings. We present a 64-year-old female with a history of type 2 diabetes mellitus and recurrent UTIs who presented to the outpatient clinic with dysuria and increased urinary frequency. Urine culture subsequently grew >100,000 CFU/mL of *R. planticola*. The patient was successfully treated with a five-day course of nitrofurantoin. This case highlights a rare instance of community-acquired UTI due to *R. planticola* in an immunocompetent outpatient. It underscores the importance of accurate microbiological identification and the consideration of atypical pathogens in patients with recurrent UTIs. This report contributes to the limited literature on *R. planticola* as a uropathogen and supports nitrofurantoin as an effective treatment option.

## Introduction

*Raoultella planticola *(*R. planticola*) is an environmental gram-negative, encapsulated, nonmotile bacterium originally classified under *Klebsiella* and reclassified as *Raoultella spp.* in 2001 [1]. It is commonly found in plants, soil, and water but is a rare source of human pathogenesis. Most reported cases of human infection usually involve an immunocompromised host [2]. A 2019 case report and review of *R. planticola* urinary tract infection (UTI) identified a total of 31 cases of human infection with *R. planticola*, only four of which involved the urinary tract [3]. Moreover, the reported cases involved patients who were hospitalized or in postoperative recovery [2,3]. This is a case of a 64-year-old immunocompetent female who was found to have a UTI due to* R. planticola* in the outpatient clinical setting.

## Case presentation

A 64-year-old female with a past medical history of hypertension, type 2 diabetes mellitus that was well controlled on insulin, hypothyroidism, and recurrent UTIs presented to the outpatient clinic with complaints of dysuria and increased urinary frequency, onset three days before the presentation. The patient denied fever, chills, flank pain, or nausea. On physical examination, she was found to be afebrile and hemodynamically stable. She experienced suprapubic tenderness to palpation, without costovertebral angle tenderness.

The urinalysis showed turbid urine with rare bacteria, but it was otherwise unremarkable without leukocytes or nitrites. The rest of the laboratory workup was remarkable for leukocytosis, with a white blood count of 14.9 K/mcL(reference values: 4.5-11.0 K/mcL) and thrombocytosis with a platelet count of 427 (reference values: 130-400 K/mcL), as shown in Table 1.

**Table 1:** Laboratory workup at the time of presentation MCV: Mean corpuscular volume; MCH: Mean corpuscular hemoglobin; MCHC: Mean corpuscular hemoglobin concentration; RDW: Red cell distribution width; MPV: Mean platelet volume; BUN: Blood urea nitrogen; eGFR: Estimated glomerular filtration rate; HPF: High-power field.

Component	Result	Reference Range
Urinalysis		
Color, urine	Yellow	Colorless-Amber
Clarity, urine	Turbid	Clear
pH, urine	5.5	5.0-9.0
Leukocytes, urine	Negative	Negative
Nitrite, urine	Negative	Negative
Protein, urine	Negative	Negative
Glucose, urine	1+	Negative
Ketones, urine	Negative	Negative
Urobilinogen, urine	0.2	0.2-1.0 mg/dL
Bilirubin, urine	Negative	Negative
Blood, urine	Negative	Negative
RBC, urine	None seen	None seen, 0-2/HPF
WBC, urine	0-5	None seen, 0-5/HPF
Bacteria, urine	Rare	None/HPF
Squamous epithelial, urine	0-5	0-5/HPF
Mucus, urine	Rare	None/HPF
Specific gravity, urine	1.018	1.003-1.035
Complete blood count		
WBC	14.9	4.5-11.0 K/mcL
RBC	5.11	3.5-5.5 K/mcL
Hemoglobin	14.0	12.0-16.0 g/dL
Hematocrit	42.1	36%-48%
MCV	82.5	80-100 FL
MCH	27.4	27-34 pcg
MCHC	33.2	30-36 g/dL
RDW	16.9	12%-15%
Platelets	427	130-400 K/mcL
MPV	7.8	6.2-12.1 FL
Basic metabolic panel		
Sodium	143	136-145 mmol/L
Potassium	4.5	3.5-5.1 mmol/L
Chloride	107	98-107 mmol/L
Serum bicarbonate	25	21-31 mmol/L
Anion gap	11	5-15
Glucose	113	74-109 mg/dL
BUN	8	7-25 mg/dL
Creatinine	0.70	0.60-1.20 mg/dL
eGFR	97	≥60 mL/min/1.73m^2^
BUN/creatinine ratio	11.4	6.0-20.0
Calcium	8.8	8.6-10.3 mg/dL

The sample was sent for further analysis, and cultures were significant for >100,000 CFU/mL *R. planticola*. Antibiotic susceptibility ​​​​​showed resistance to trimethoprim/sulfamethoxazole and ampicillin and sensitivity to ampicillin/sulbactam, levofloxacin, and nitrofurantoin (Table 2). The patient was successfully treated with a five-day course of nitrofurantoin. Upon outpatient follow-up six weeks later, the patient reported complete resolution of symptoms.

**Table 2:** Antibiotic susceptibility of Raoultella planticola in urine culture

Antibiotic	Susceptibility
Ampicillin	Resistant
Ampicillin/Sulbactam	Susceptible
Gentamicin	Susceptible
Levofloxacin	Susceptible
Nitrofurantoin	Susceptible
Tobramycin	Susceptible
Trimethoprim/Sulfamethoxazole	Resistant

## Discussion

This case is notable for being one of the few documented instances of community-acquired UTI caused by *R. planticola* in an outpatient setting [3]. Unlike the majority of previously reported cases, which occurred in hospitalized, postoperative, or immunocompromised patients [2,3], our patient had no recent hospitalizations, no urinary instrumentation, and no history of immunosuppression. Her only identified risk factor was well-controlled type 2 diabetes mellitus, a known contributor to increased susceptibility to UTIs and previously suggested as a predisposing factor for *R. planticola* infections [4]. It is important to note that her laboratory workup demonstrated leukocytosis, but her urine sample did not report leukocytes. This discrepancy was most likely due to the technical and interpretive limitations of this test. This remark highlights how valuable proper history-taking is in leading to further testing, such as urine culture.

Recent literature has expanded on the role of *R. planticola* in UTIs. In a case reported by Skelton et al., a 73-year-old immunocompromised woman with a history of multiple myeloma presented with loose stools and hemodynamic instability ultimately attributed to *R. planticola* [5]. Urine culture sensitivity analysis demonstrated resistance to ampicillin, cefazolin, ceftazidime, ceftriaxone, gentamicin, and tobramycin and sensitivity to trimethoprim-sulfamethoxazole. There is a contrast with our case, in which the isolated strain demonstrated resistance to both ampicillin and trimethoprim-sulfamethoxazole, reinforcing the importance of tailoring therapy to sensitivity profiles rather than relying on standard empiric regimens, since these can change. It is important to note that our patient was not immunocompromised.

Furthermore, emerging epidemiological data suggest that *Raoultella *species may behave similarly to multidrug-resistant *Klebsiella*, with a growing potential to become clinically significant pathogens in both hospital and community settings. In a retrospective study by Alp et al., the most common *R. planticola*-related infections were bloodstream infections, particularly in patients with indwelling catheters or those in the intensive care unit (ICU) [6]. This contrasts sharply with our patient, who was managed entirely in the outpatient setting, had no invasive devices, and remained hemodynamically stable throughout. These findings underscore the importance of recognizing *R. planticola* as a potential emerging pathogen even outside the ICU or nosocomial context.

More severe presentations have also been documented. Yumoto et al. reported a fatal case of *R. planticola* bacteremia in a 79-year-old man who developed septic shock 10 days after suffering extensive flame burns [7]. Despite appropriate antibiotic therapy, the patient died within two days of the onset of sepsis. Although this case is significantly different in both severity and context from our patient, it illustrates the pathogenic potential of *R. planticola *in vulnerable hosts and raises concerns about the organism’s virulence under certain conditions [8]. Fortunately, our outpatient case followed a benign course, but clinicians should be mindful of *R. planticola*'s potential to cause systemic and life-threatening illness.

Surgical site infections have also been linked to *R. planticola*, as highlighted in a case described by Tufa et al., in which the organism was isolated from an infected wound following repeated abdominal surgery [9]. The authors emphasized that *R. planticola* may be an underrecognized cause of nosocomial infections with high levels of antimicrobial resistance. Although our patient had no surgical history or recent antibiotic exposure, the report provides valuable insights into the broader spectrum of *R. planticola* infections and highlights its increasing relevance in both community-acquired and nosocomial settings. For community-acquired settings, there is one bacteremia case reported in the setting of enteric fever-like syndrome caused by seafood consumption [10]. In this case, the strain was pan-sensitive.

Taken together, these cases, along with ours, support the need for improved awareness of *R. planticola* as an emerging opportunistic pathogen. Clinicians should maintain a high index of suspicion, particularly in patients with recurrent UTIs or underlying comorbidities such as diabetes mellitus. Importantly, this case reinforces the importance of culture-guided therapy, as *R. planticola* may display resistance to commonly used empiric agents, including trimethoprim-sulfamethoxazole and ampicillin [10]. Given its phenotypic similarity to *Klebsiella spp*., misidentification remains a concern, potentially leading to inappropriate therapy. As such, accurate microbiologic identification and antimicrobial susceptibility testing are essential to optimize outcomes.

To our knowledge, this represents one of the rare, if not the first, documented cases of *R. planticola* UTI managed entirely in an outpatient setting. It contributes to the growing body of evidence that* R. planticola* infections are not restricted to critically ill or hospitalized patients and should be considered in the differential diagnosis of UTIs, especially in patients with recurrent presentations and underlying risk factors.

## Conclusions

*R. planticola* is a rare organism that usually affects immunocompromised patients, but it is an emerging pathogen that may cause disease in immunocompetent patients. Accurate microbiological identification and the consideration of atypical pathogens in patients with recurrent UTIs are crucial to guide therapy. This report contributes to the limited number of cases in the literature on *R. planticola* as a uropathogen.
